# Exogenous HIV-1 Nef Upsets the IFN-γ-Induced Impairment of Human Intestinal Epithelial Integrity

**DOI:** 10.1371/journal.pone.0023442

**Published:** 2011-08-08

**Authors:** Maria Giovanna Quaranta, Olimpia Vincentini, Cristina Felli, Francesca Spadaro, Marco Silano, Diego Moricoli, Luciana Giordani, Marina Viora

**Affiliations:** 1 Department of Therapeutic Research and Medicine Evaluation, Istituto Superiore di Sanità, Roma, Italy; 2 Department of Veterinary Public Health and Food Safety, Istituto Superiore di Sanità, Roma, Italy; 3 Department of Cell Biology and Neurosciences, Istituto Superiore di Sanità, Roma, Italy; 4 Diatheva s.r.l., Fano (PU), Italy; Institut Pasteur, France

## Abstract

**Background:**

The mucosal tissues play a central role in the transmission of HIV-1 infection as well as in the pathogenesis of AIDS. Despite several clinical studies reported intestinal dysfunction during HIV infection, the mechanisms underlying HIV-induced impairments of mucosal epithelial barrier are still unclear. It has been postulated that HIV-1 alters enterocytic function and HIV-1 proteins have been detected in several cell types of the intestinal mucosa. In the present study, we analyzed the effect of the accessory HIV-1 Nef protein on human epithelial cell line.

**Methodology/Principal Findings:**

We used unstimulated or IFN-γ-stimulated Caco-2 cells, as a model for homeostatic and inflamed gastrointestinal tracts, respectively. We investigated the effect of exogenous recombinant Nef on monolayer integrity analyzing its uptake, transepithelial electrical resistance, permeability to FITC-dextran and the expression of tight junction proteins. Moreover, we measured the induction of proinflammatory mediators. Exogenous Nef was taken up by Caco-2 cells, increased intestinal epithelial permeability and upset the IFN-γ-induced reduction of transepitelial resistance, interfering with tight junction protein expression. Moreover, Nef inhibited IFN-γ-induced apoptosis and up-regulated TNF-α, IL-6 and MIP-3α production by Caco-2 cells while down-regulated IL-10 production. The simultaneous exposure of Caco-2 cells to Nef and IFN-γ did not affect cytokine secretion respect to untreated cells. Finally, we found that Nef counteracted the IFN-γ induced arachidonic acid cascade.

**Conclusion/Significance:**

Our findings suggest that exogenous Nef, perturbing the IFN-γ-induced impairment of intestinal epithelial cells, could prolong cell survival, thus allowing for accumulation of viral particles. Our results may improve the understanding of AIDS pathogenesis, supporting the discovery of new therapeutic interventions.

## Introduction

The gastrointestinal (GI) tract represents the largest mucosal surface in the human body. Mucosal surfaces are separated from the outside world by epithelial barriers. By blocking the passive movement of commensal bacteria, pathogens and toxins into the subepithelial environment, epithelial cells (EC) prevent the onset of local and systemic inflammation and provide a first line of defence against infection. The gut barrier is formed by tight junctions (TJ) linking adjacent EC. TJ disruption can cause increased permeability, leading to inflammatory conditions in the mucosa [1].

Pathological changes in the GI tract represent a characteristic feature of HIV infection. More than 85% of HIV infections are acquired by mucosal transmission, and quantitative and qualitative defects of mucosal immunity are present in all stages of infection [2]. The GI tract is a major site of HIV replication, resulting in massive depletion of lamina propria CD4^+^ T cells during acute infection. Chronic HIV infection is characterized by increased intestinal permeability and enteropathy, and chronic activation of the immune system, which is a significant predictor of disease progression. During the progression of the disease, chronic diarrhoea, dehydration, and malabsorption, lead to progressive weight loss, contributing to the morbidity and mortality of HIV-1^+^ subjects [3], [4].

The pathophysiology of HIV-1-related intestinal dysfunction has been attributed to opportunistic infections, cytokine secretion in response to chronic inflammation, and a direct role of HIV itself [5], [6]. HIV triggers the local release of cytokines that lead to impairment of barrier function by altering the expression of TJ-associated proteins and by inducing apoptosis of EC. The resultant barrier defect facilitates the microbial antigen translocation that further stimulates mucosal cytokine production and systemic immune activation [7]. Some effects induced by HIV-1 are mediated by viral factors, such as gp120 that accelerates human lamina propria T cell apoptosis [8]. *In vitro* studies demonstrated that Tat protein is directly involved in AIDS-associated intestinal dysfunction, affecting the uptake of glucose by enterocytes and causing microtubules depolymerisation [9], [10].

HIV-1 Nef protein is an essential factor for efficient viral replication and pathogenesis [11]. Nef exerts pleiotropic effects interfering with cellular signal transduction pathways [12]. Nef targets cell membranes and elicits cytoskeletal rearrangement, organelle formation and synapse destabilization [13]-[15]. To date, most of Nef's functions have been associated with its biochemical activities within the producer cell. However, Nef is known to be secreted from infected cells [16] in association with small membrane-bound vescicles [17], [18]. We have previously demonstrated that purified exogenous Nef enters in monocyte-derived dendritic cells (MDDC) inducing their activation. This has a direct impact on CD4^+^ T cell bystander activation and on the impairment of CD8^+^ T and NK cell function [19]–[21]. Moreover, exogenous Nef downregulates the induction of specific antibody responses [22].

Despite numerous reports describe how HIV affects mucosal immunity, the pathobiology of Nef in mucosal dysfunction remains unknown. In the present study, we investigated the effect of HIV-1 Nef exposure on intestinal epithelial cells, using Caco-2 cell line, representing the best *in vitro* model currently available of human enterocytes able to differentiate spontaneously in long term culture.

We analyzed the effect of HIV-1 Nef on both monolayer integrity and induction of proinflammatory mediators. We demonstrated that exogenous Nef was taken up by Caco-2 cells, increased intestinal epithelial tight junction permeability and upset the IFN-γ-induced impairment of intestinal epithelial cells.

## Materials and Methods

### Nef protein

Recombinant HIV-1 Nef (BRU variant) was expressed in E.coli and purified to homogenity by ionic exchange and size exclusion chromatography from DIATHEVA s.r.l (Fano-ITALY). The Nef protein was highly purified (>99%) as assessed by SDS-PAGE, Western Blotting, and by analytical HPLC. Lyophilized protein was dissolved in sterile water and aliquots were stored at -70°C. The biological activity was assessed by induction of phenotypical and functional activation of MDDC [19]. Endotoxin content of Nef, determined by Pyrotell Limulus amebocyte Lysate assay (Cape Cod Inc. Falmouth MA. USA) was <0.03 unit/ml.

A dose-response titration curve was performed to assess the optimal concentrations of Nef. In all subsequent experiments Nef was used at 0.1 µg/ml.

In some experiments Nef was preincubated with 10 µg/ml anti-Nef monoclonal antibody (mAb) or with 10 µg/ml anti-Tat mAb (DIATHEVA s.r.l). for 60 min at 37°C. Moreover, to further exclude any contamination due to bacterial expression or non-specific effects of Nef, boiled Nef (bNef; 100° C, 10 min) or Nef_F12_ were used as negative controls. The recombinant Nef_F12_ protein (kindly provided by M. Federico, National AIDS Center, Istituto Superiore di Sanità, Rome, Italy), a full-length Nef mutant lacking the most relevant Nef functions [23], [24], was recovered and purified as previously described [25].

Cell culture supernatants containing Nef were kindly provided by M. Federico, National AIDS Center, Istituto Superiore di Sanità, Rome, Italy. Supernatants were obtained by transfecting 293T cells with either a pcDNA3.1 vector expressing wt Nef HIV-1, strain NL4-3, or, as control, with the vector alone. Two days later, the supernatants were collected, clarified, and then subjected to ten-fold concentration using a Centricon device, cut-off 20 kDa, and forced dialysis against 1× PBS. The amount of Nef in concentrated supernatants was estimated by semi-quantitative anti-Nef Western blot assay against quantified amount of recombinant Nef, which was carried out as already described [26].

### Caco-2 cell monolayers

Caco-2 human colonic carcinoma cells were maintained in 5% CO_2_ at 37°C in Dulbecco's modified essential medium (DMEM, Lonza, Verviers, Belgium) containing 4.5 g/L glucose, 2 mM L-glutamine, 50 U/mL penicillin, 50 µg/mL streptomycin, 1% nonessential amino acids, 1% HEPES and 10% heat-inactivated fetal bovine serum (FBS, Lonza), as described previously [27]. Cells were allowed to differentiate for 21 days and the medium was regularly changed three times per week.

### Permeability measurement

Caco-2 cells were seeded at a density of 4.4×10^4^ cells/cm^2^ on polycarbonate inserts (0.4 µm pore diameter, 0.9 cm^2^ area). After 21 days differentiated cells were treated from the basolateral compartment with Nef (0.01-0.1-1 µg/ml), IFN-γ (BD Biosciences, Bedford, MA; 600 U/ml), IFN-γ/Nef or with supernatants of trasfected 293T cells. Boiled Nef (0.1 µg/ml) and Nef_F12_ (0.1 µg/ml) were used as negative controls.

Fluorescein isothiocyanate-conjugated dextran (FITC-dextran; MW, 4.4 kd; Sigma, St. Luis, MO) was dissolved in culture medium and used at a final concentration of 2.2 mg/ml in the apical cell compartment. After 3 h of incubation the amount of fluorescence was measured in the basal compartment with a spectrofluorometer. The excitation and emission wavelengths were 490 and 520 nm, respectively.

### Transepithelial electrical resistance of Caco-2 cells

Caco-2 cells were grown and left to differentiate for 21 days on 0.4 µm pore inserts and transepithelial electrical resistance (TEER) of monolayers was measured using a Millicell-ERS device (Millipore, Bedford, MA, USA). The results were normalized with the values of TEER measured before treatment, subtracting from each point the TEER value of the filter alone. TEER data were recorded at room temperature 24, 48 and 72 h after treatment with Nef, IFN-γ or their combination.

### Western blot analysis

Cells were resuspended into a buffer containing 150 mM NaCl, 0.01% sodium dodecylsulfate (SDS) and 1% Triton X-100 and then centrifuged for 5 min at 4°C. The protein concentration was measured by the Laemmli method, using the Bio-Rad protein assay with BSA as standard (Biorad, Hercules, CA). SDS-polyacrylamide gel electrophoresis was carried out on 4% stacking and 7.5% resolving gel. 30 µg of proteins were loaded in each lane with loading buffer containing 0.1Tris (pH 6.8), 20% glycerol, 10% mercaptoethanol, 4% SDS and 0.2% Bromophenol Blue. Following electrophoresis, the proteins were transferred to a nitrocellulose membrane (Biorad). Membranes were blocked for 1 h with 5% non-fat milk in TBST (100 mM NaCl, 5 mM KCl, 100 mM Tris-HCl, pH 7.4 and 0.05% Tween 20) and probed with primary mouse IgG monoclonal antibody (mAb) against occludin (clone OC-3F10, Zymed, San Francisco, USA), mouse IgG mAb against ZO-1 (clone 1/ZO-1, BD Biosciences) or mouse IgG mAb against cyclooxygenase (COX)-2 (Abcam, Cambridge, UK) for 24 h at +4°C. Secondary antibody (goat-anti-mouse conjugated to horse-radish peroxidase HRP; Biorad) was added at a 1∶3000 dilution for 1 h at room temperature. Proteins were revealed by the chemiluminescence detection kit (Biorad), according to manufacturer's instructions. Intensities of protein bands were evaluated using the Biorad ChemiDoc densitometer. Membranes were stripped and reprobed with primary rabbit mAb against β-actin (Sigma) and then secondary goat-anti rabbit-HRP (Biorad).

### Fluorescence microscopy

Cellular localization of ZO-1 was assessed by immunofluorescence analysis. Caco-2 cells were grown on glass coverslips and after 21 days cells were treated for 48 h with Nef, IFN-γ or IFN-γ/Nef. Cells were then fixed with PBS/3.7% formaldehyde (30 min, room temperature), permeabilized with 0.5% (v/v) Triton X-100 (Sigma) in PBS (5 min, room temperature) and stained with mouse anti-ZO-1 mAbs (BD Biosciences) at 37°C for 30 min. Cells were then incubated with anti-mouse IgG fluorescein-linked whole antibody (Sigma) at 37°C for 30 min. All samples were mounted with glycerol:PBS (2∶1) and analyzed by fluorescence with a Nikon Microphot microscope (Carl Zeiss, Germany) using SCANware (Leica Lasertecnik GMBH) and Photoshop (Adobe Sistems Inc) software.

### Confocal laser scanning microscopy (CLSM)

For Nef entry detection, Caco-2 cells were seeded on polycarbonate inserts and after 21 days were treated basolaterally with biotynilated-Nef (DIATHEVA s.r.l) in the presence or absence of IFN-γ for 1, 3, 6 and 24 h. After treatments the inserts were cut, washed in PBS, fixed with PBS/3% paraformaldehyde (30 min at 4°C), permeabilized with 0.5% (v/v) Triton X-100 (Sigma) in PBS (10 min, room temperature). Then, the inserts were stained with avidin-FITC (BioLegend, San Diego, CA) at 37°C for 30 min. All samples were finally mounted on the microscope slide with Vectashield antifade mounting medium containing 4′,6′-diamidino-2-phenylindole (DAPI, Vector Laboratories, Peterborough, UK). CLSM observations were performed with a Leica TCS SP2 AOBS apparatus, using excitation spectral laser lines at 405 and 488 nm and using the Leica Confocal Software (Leica Lasertechnik) and Adobe Photoshop software programs (Adobe system Incorporated). Several cells were analysed for each labelling condition and representative results are presented.

### Apoptotis detection

Annexin V assay was used to detect Caco-2 cell apoptosis. After 48 h of Nef, IFN-γ or IFN-γ/Nef treatment, Caco-2 cells were harvested and washed twice with ice cold PBS and specific binding of FITC-conjugate annexin V/propidium iodide was performed with an apoptosis detection kit (Bender, Wien, Austria) accordingly to the manufacturer's instructions. The cells were then analysed with a FACScan flow cytometer and CellQuest software (BD Biosciences) to measure co-staining of annexin V and propidium iodide.

### Cytokine ELISA

Supernatants from the basolateral chambers were collected 48 h after treatment with Nef, IFN-γ or their combination and IL-6, IL-10, TNF-α, MIP-3α and IL-8 secretion was measured by sandwich ELISA (BD OptEIA, San Diego, CA for IL-6, IL-10, TNF-α; R&D Systems, Abingdon, UK for MIP-3α; Bender for IL-8) according to the manufacturer's instructions.

### Cytoplasmic phospholipase A2 (cPLA_2_) enzyme activity assay

cPLA_2_ activity was assessed in Caco-2 cell lysates by the cPLA_2_ assay kit (Cayman Chemicals, Ann Arbor, MI) according to the manufacturer's instructions. In brief, cells were treated with Nef, IFN-γ or INF-γ/Nef for 4 h and harvested using a rubber policeman. The cell pellets were sonicated in cold PBS. 10 µl sample supernatant and 5 µl assay buffer were well mixed and the reaction was initiated by adding 200 µl substrate solution to the wells. After 1 h incubation at room temperature, 10 µl of DTNB/EGTA was added to each well to stop the reaction. Absorbance was measured at 414 nm.

### Prostaglandin E_2_ (PGE_2_) quantification

PGE_2_ concentration was determined in the culture medium of differentiated Caco-2 monolayers after 24 h of Nef, IFN-γ or INF-γ/Nef treatment. PGE_2_ levels were determined with a PGE_2_-monoclonal enzyme immunoassay kit (Cayman Chemicals) following the manufacturer's protocol.

### Statistical Analysis

Statistical significance of differences was calculated using a Student's t test. A p value of less than 0.05 was considered as statistically significant.

## Results

### HIV-1 Nef modulates the permeability of intestinal epithelial monolayers

**Figure 1:**
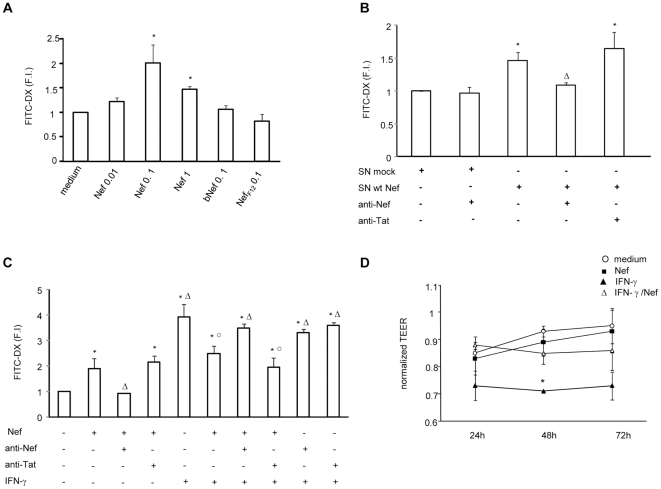
Effect of HIV-1 Nef on paracellular flux of FITC-dextran and transepithelial electrical resistance (TEER) in untreated or IFN-γ treated intestinal epithelial cell monolayer. (A) Caco-2 cells were grown and differentiated on inserts and then left untreated (medium) or treated with graded amounts of Nef (ranging from 0.01 to 1 µg/ml), boiled Nef (0.1 µg/ml, bNef) or Nef_F12_ (0.1 µg/ml) for 48 h. FITC-dextran (FITC-DX) was added in the apical compartment and after 3 h, the fluorescence intensity was measured in the basolateral compartment. Data represent means ± SEM of 3 independent experiments in duplicate. F.I. fold increase. (B) Caco-2 cells were treated for 48 h with supernatants from 293T cells transfected with either a pcDNA3.1 vector expressing wt Nef HIV-1 (SN wt Nef, containing 0.01 µg/ml Nef), or with the vector alone (SN mock). The amount of Nef in supernatants was estimated by semi-quantitative anti-Nef Western blot assay against quantified amounts of recombinant Nef. (C) Caco-2 cells were left untreated or treated with Nef (0.1 µg/ml), IFN-γ (600 U/ml) or their combination for 48 h. Nef and IFN-γ were preincubated with an anti-Nef mAb (10 µg/ml) or anti-Tat mAb (10 µg/ml) for 1 h at 37°C and then added to Caco-2 cells for 48 h. Means ± SEM from 4 independent experiments in duplicate, are shown. (D) Caco-2 cells were cultured on filter inserts for 21 days and then left untreated (medium ○) or treated with Nef (0.1 µg/ml ▪), IFN-γ (600 U/ml ▴) or IFN-γ/Nef (Δ). The TEER values were measured at the indicated time points and were normalized with values obtained at the start point of the analysis. Data represent means ± SEM of 8 independent experiments in duplicate. *p<0.05 vs medium; ^○^p<0.05 vs IFN-γ; ^Δ^p<0.05 vs Nef.

Since HIV-1 infection is associated with increased permeability of the intestinal tract, we first evaluated whether HIV-1 Nef affects the permeability of polarized epithelial cells. To this aim, graded doses of exogenous Nef were added basolaterally to confluent monolayers of differentiated Caco-2 cells grown in transwells, for 48 h. To test whether Nef impacts on monolayer permeability, FITC-dextran was added in the apical compartment and after 3 h the fluorescence intensity was measured in the basolateral compartment. As shown in Fig. 1A, untreated Caco-2 cell monolayers were not readily permeable to FITC-dextran while Nef significantly up-regulated epithelial monolayer permeability, showing the highest tracer flux at 0.1 µg/ml. Boiled Nef and Nef_F12_, used as negative controls, did not modulate epithelial monolayer permeability, confirming that the observed effect was not due to bacterial expression or purification procedure.

In a second set of experiments we examined whether the significative effect obtained with the recombinant Nef protein can be confirmed with a lower dose of native Nef protein, using supernatants from Nef-transfected 293T cells. Interestingly, we found that supernatants from trasfected cells, containing 0.01 µg/ml Nef, significantly up-regulated epithelial monolayer permeability respect to supernatants of mock-transfected 293T cells (Fig. 1B).

Since the advanced stages of HIV infection are characterized by chronic inflammation of the GI tract, we evaluated the effect of Nef on Caco-2 cells exposed to IFN-γ inflammatory mediator (Fig. 1C). As expected, upon 48 h treatment with IFN-γ the monolayer become more permeable showing a significant increase in the tracer flux respect to untreated Caco-2 cells. Notably, Nef partially abrogated the effect of IFN-γ, showing a significant reduction in the tracer flux respect to IFN-γ. Preincubation of Nef with anti-Nef mAb, but not with anti-Tat mAb, abrogated the effect of Nef, confirming that the Nef-induced modulation of monolayer permeability was indeed a Nef-specific effect.

Next, to quantify the movement of ions across the paracellular pathway, we evaluated the effect of Nef, IFN-γ or their combination, on TEER of Caco-2 cell monolayer. As shown in Fig. 1 D, Nef did not significantly modulate the TEER, while IFN-γ reduced the TEER at all times analyzed, and significantly at 48 h (p<0.05) compared to untreated cells (medium). As already observed in tracer flux assay, the addition of Nef abrogated the effect of IFN-γ at all times analyzed, maintaining TEER to levels similar to those of untreated cells.

### HIV-1 Nef affects the expression and localization of tight junction proteins

**Figure 2:**
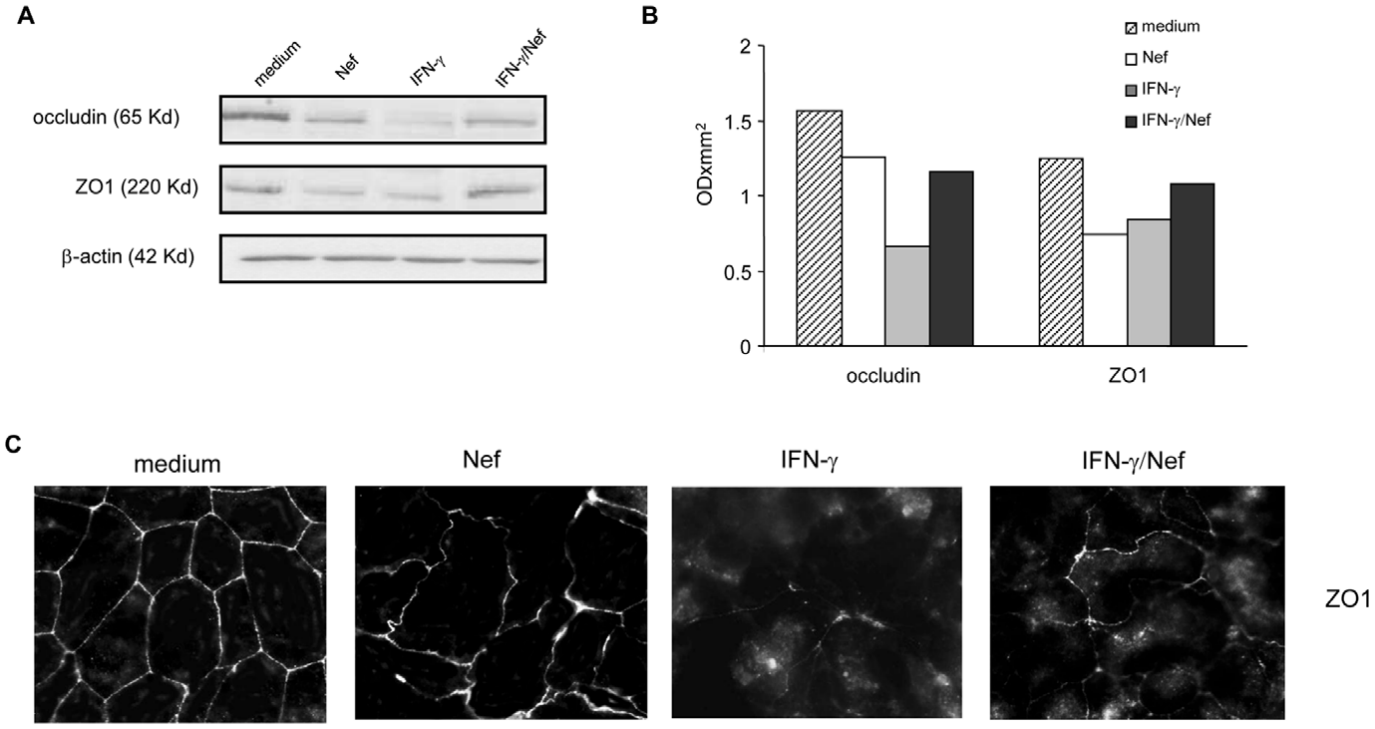
Effect of HIV-1 Nef on tight junction protein expression and localization in untreated or IFN-γ treated intestinal epithelial cell monolayer. (A) Differentiated Caco-2 cells were left untreated (medium) or were treated for 48 h with Nef, IFN-γ or their combination. Cells were then lysed and proteins were probed on Western blots with anti-occludin or anti-ZO1 mAbs. (B) The expression of the proteins was estimated by densitometry after normalization with the β-actin signal. Data represent values from 1 experiment representative of 3 performed. (C) Immunofluorescence analysis of ZO1 distribution in untreated (medium) or 48 h Nef-, IFN-γ - or IFN-γ/Nef- treated Caco-2 monolayers. The experiment was repeated 3 times to ensure reproducibility.

Since TJ regulate paracellular permeability, we examined the effect of Nef, IFN-γ or their combination on the expression of two TJ proteins, ZO1 and occludin. To this aim, Caco-2 cells were left untreated or were treated with Nef, IFN-γ or their combination and immunoblot analyses were performed after 48 h. Densitometric analysis shows that Nef induced a 1.25- and 1.7-fold reduction in occludin and ZO1 expression level respectively, while IFN-γ induced a 2.5- and 1.5-fold reduction, respectively. Notably, using the combination, occludin and ZO1 expression levels were comparable to that obtained in lysates from Nef-treated monolayers (Fig. 2A,B).

Next, we analysed the cellular localization of ZO1 protein by immunofluorescence (Fig. 2C). As expected, localization of ZO1 showed strong peripheral labelling in untreated Caco-2 cells. Nef treatment reduced the intensity of ZO1 staining at cell-to-cell contacts. No increase of immunofluorescence signal was observed inside the cells, confirming that Nef down-regulated ZO-1 expression. Furthermore, Nef-treated cells appeared morphologically altered with irregular contours. Treatment of monolayers with IFN-γ caused a disturbance in the continuity of ZO1 localization at the cellular borders; the intensity of staining for ZO1 was also reduced and intracellular pools of ZO1 were apparent. To be noted that Nef partially prevented the IFN-γ induced alteration in junctional localization and reduction of ZO1 protein.

### HIV-1 Nef is taken up by Caco-2 cells

**Figure 3:**
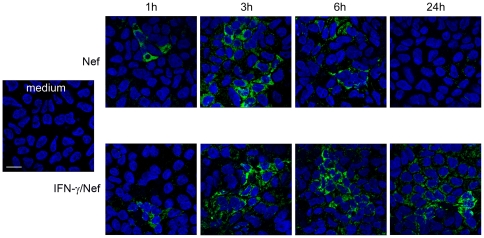
HIV-1 Nef uptake in intestinal epithelial cells. Differentiated Caco-2 cells were left untreated (medium) or were treated for 1, 3, 6 and 24 h with biotynilated-Nef alone or in combination with IFN-γ. Nef uptake was analyzed by CLSM (central optical sections). Nuclei are reported in blue (DAPI). Nef was detected as a diffuse intracytoplasmatic positivity (green). Scale bar, 20 µm. Panels are representative of 3 independent experiments.

The uptake of Nef by Caco-2 cells was monitored after 1, 3, 6 and 24 h of treatment in absence or presence of IFN-γ. As shown in Fig. 3 Nef uptake was already detectable after 1 h of treatment, independently from IFN-γ treatment, appearing as a diffuse intracytoplasmatic positivity. In the absence of IFN-γ, the level of intracellular staining reached the higher brightness after 3 h of treatment, declining with time and virtually disappearing after 24 h. Interestingly, in IFN-γ-treated Caco-2 cells, the level of internalized Nef was substantially unaltered from 3 h up to 6 h, and an intense fluorescence was still retained after 24 h of treatment.

### HIV-1 Nef inhibits IFN-γ-induced apoptosis

**Figure 4:**
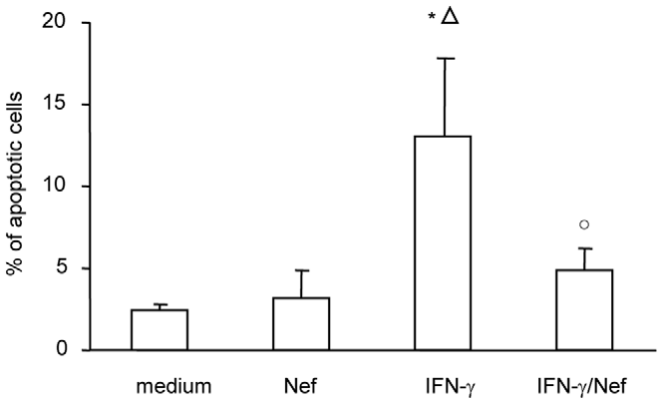
Effect of HIV-1 Nef on untreated or IFN-γ treated intestinal epithelial cell apoptosis. Differentiated Caco-2 cells were left untreated (medium) or were treated for 48 h with Nef, IFN-γ or their combination. Quantitative evaluation of apoptosis was performed by flow cytometry measuring co-staining of annexin V and propidium iodide. Means ± SEM from 5 independent experiments in duplicate, are shown. *p<0.05 vs medium; ^○^p<0.05 vs IFN-γ; ^Δ^p<0.05 vs Nef.

Since IFN-γ is known to compromise barrier function and to induce apoptosis [28], we examined whether Nef, alone or in combination with IFN-γ, affects Caco-2 cell apoptosis. As shown in Fig. 4, exposure of monolayers to Nef for 24 h did not induce Caco-2 cell apoptosis compared to untreated Caco-2 cells. As expected, incubation with IFN-γ significantly increased the percentage of apoptotic cells respect to untreated and Nef-treated Caco-2 cells. Interestingly, co-incubation of IFN-γ and Nef did not increase the percentage of apoptotic cells respect to untreated cells.

### HIV-1 Nef affects cytokine and chemokine production by Caco-2 cells

In response to tissue damage, infection or exposure to antigens, EC release inflammatory cytokines that can directly affect mucosal integrity. We therefore examined the cytokine secretion profile of Caco-2 cells after 48 h of treatment with Nef, IFN-γ or their combination.

**Figure 5:**
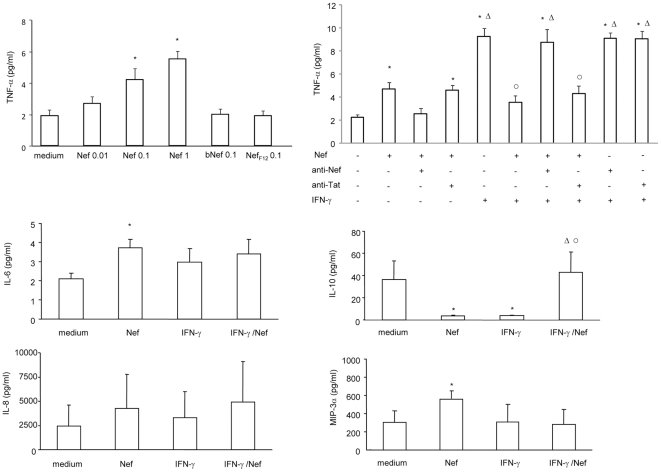
Effect of HIV-1 Nef on cytokine and chemokine secretion by untreated or IFN-γ treated intestinal epithelial cells. Caco-2 cells were grown and differentiated on inserts and then left untreated (medium) or treated with graded amounts of Nef (ranging from 0.01 to 1 µg/ml), boiled Nef (0.1 µg/ml, bNef) or Nef_F12_ (0.1 µg/ml). After 48 h supernatants were harvested from the basolateral chambers and tested for cytokines (TNF-α, IL-6, IL-10) and chemokines (MIP-3α, IL-8) by ELISA. In some experiments Nef and IFN-γ were preincubated with an anti-Nef mAb (10 µg/ml) or anti-Tat mAb (10 µg/ml) for 1 h at 37°C and then added to Caco-2 cells for 48 h. Results are expressed as pg/ml and are means ± SEM from 4 independent experiments in duplicate. *p<0.05 vs medium; ^○^p<0.05 vs IFN-γ; ^Δ^p<0.05 vs Nef.

As shown in Fig. 5, Nef significantly up-regulated TNF-α secretion in a dose dependent manner, while bNef and Nef_F12_ did not exert any effect. Preincubation of Nef with anti-Nef mAb, but not with anti-Tat mAb, abrogated the effect of Nef used alone or in combination with IFN-γ.

In addition, following Nef treatment there was a significant increase in IL-6 and MIP-3α release, while IL-10 was significantly down-regulated.

Exposure of Caco-2 cells to IFN-γ induced a significant up-regulation of TNF-α production and a significant down-regulation of IL-10, while did not alter IL-6, MIP-3α and IL-8 secretion.

Notably, Nef abrogated the IFN-γ-induced up-regulation of TNF-α production and the IFN-γ-induced down-regulation of IL-10 production. The simultaneous exposure to Nef and IFN-γ did not affect cytokine and chemokine secretion respect to untreated cells.

### HIV-1 Nef inhibits the IFN-γ-induced arachidonic acid cascade

Since arachidonic acid-derived eicosanoids exert important roles in immunophatology and in inflammation, we examined whether Nef, alone or in combination with IFN-γ, interferes with arachidonic acid (AA) cascade.

**Figure 6:**
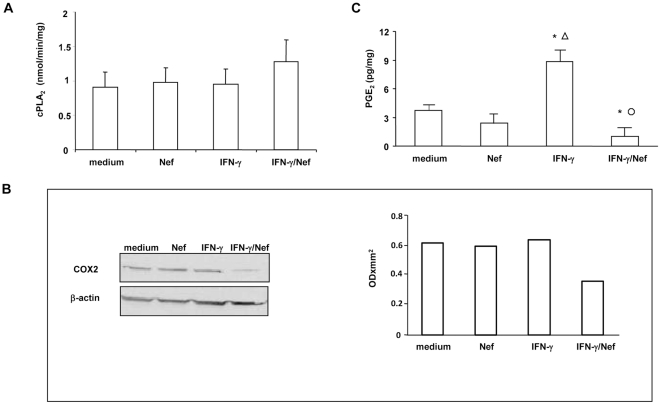
Effect of HIV-1 Nef on arachidonic acid cascade in untreated or IFN-γ treated intestinal epithelial cells. Differentiated Caco-2 cells were left untreated (medium) or were treated with Nef, IFN-γ or their combination. (A) After 4 h cPLA_2_ enzyme activity was assessed in cell lysates. Results are expressed as nmol/min/mg protein and are means ± SEM from 5 independent experiments in duplicate. (B) After 24 h, proteins were probed on Western blots with anti-COX-2 polyclonal antibody. The expression of COX-2 was estimated by densitometry after normalization with the β-actin signal. Blots are representative of three independent experiments. (C) After 24 h cell supernatants were harvested and PGE_2_ concentrations were measured by ELISA, according the manufacturer's instruction. Results are expressed as pg/ml and are means ± SEM from 4 independent experiments in duplicate. *p<0.05 vs medium; ^○^p<0.05 vs IFN-γ; ^Δ^p<0.05 vs Nef.

As shown in Fig. 6A, neither Nef nor IFN-γ or their combination modulated the activity of cPLA_2_, a key enzyme in prostaglandin production, respect to untreated cells, after 4 h of treatment.

We then evaluated the expression of COX-2, the enzyme involved in the conversion of arachidonic acid to prostaglandin. The results obtained indicate that Nef and IFN-γ did not modulate COX-2 expression after 24 h of treatment. Interestingly, co-incubation of IFN-γ and Nef down-regulated COX-2 expression respect to untreated, Nef- or IFN-γ-treated cells (Fig. 6B).

Finally, we measured the biologically active product PGE_2_, after 24 h of Nef, IFN-γ or IFN-γ/Nef treatment. We found that Nef did not affect PGE_2_ release. On the other hand, IFN-γ significantly up-regulated PGE_2_ release. In agreement with COX-2 reduction, Nef abrogated the IFN-γ-induced up-regulation of PGE_2_ release (Fig. 6C).

## Discussion

In the present study, we demonstrate that exogenous HIV-1 Nef increases intestinal epithelial tight junction permeability and abrogates the effects induced by IFN-γ on intestinal EC monolayer permeability and cell viability inhibiting the IFN-γ induced arachidonic acid cascade.

We investigated the effect of Nef exposure on Caco-2 cell line, as a model for GI tracts. Caco-2 cells undergo a gradual differentiation process that takes place spontaneously once confluence has been reached, characterized by the acquisition of morphological and functional polarity highly comparable to those of mature enterocytes. Caco-2 cells express functional tight junctions, brush border characteristics and biotrasformation enzymes, and their differentiation process is thought to reflect the maturation occurring *in vivo* along the crypt-villus axis, which makes Caco-2 probably the best *in vitro* model of human enterocytes currently available [29].

Increased mucosal inflammation is an important feature of acute HIV infection and is associated with lower systemic CD4^+^ T cell counts. HIV-1 in line with active intestinal bowel disease (IBD) is associated with a strong inflammatory response in the gut lamina propria and a number of parallels are observed between HIV and IBD in terms of gut disease, such as an increased intestinal permeability and enteropathy [3]. IFN-γ accurately mimics intestinal inflammatory process and a number of published studies employ this cytokine as a valid and consistent *in vitro* model of the physio-pathological behaviour of enterocytes during the acute phase of IBD [28], [30], [31]. IFN-γ is a major interplayer of inflammation and immune responses, and has been reported to have suppressive/enhancing effects on HIV which may depend on cell type or activation status [32]-[34]. Thus, in the present study, we analyzed the effect of Nef on both unstimulated and IFN-γ-stimulated Caco-2 cells, to mimic the early phase of infection and the inflammatory acute phase. We found that IFN-γ reduced transepithelial resistance, a measure of monolayer integrity. The decrease in TEER correlated with increased permeability and disruption of tight junction proteins. Our data confirm previous observations that clearly established that proinflammatory cytokines are implicated in damage to the intestinal/epithelial barrier function [28], [30], [31]. Similarly, but to a lesser extent, Nef increased intestinal epithelial tight junction permeability. Interestingly, the IFN-γ-induced impairment of monolayer integrity was partially restored when Caco-2 cells are challenged simultaneously with Nef and IFN-γ, probably due to mutual cross-regulation of signalling pathways. How Nef attenuates IFN-γ-driven pathways in Caco-2 cells awaits clarification.

Nef was not able to restore the impairment of epithelial barrier integrity induced by high dose of IFN-γ (1000 U/ml) (data not shown). In addition, Nef did not exert any effect if Caco-2 cell monolayer was pre-treated with IFN-γ (data not shown). Thus, it might be assumed that Nef acts differently during the early, acute and chronic phases of HIV-infection, characterized by different inflammatory cytokine profile.

Despite numerous reports describe how HIV-1 Nef affects immune cells, the effect of Nef on EC remains unknown. In this context, we have previously demonstrated that exogenous Nef regulates co-receptor expression and cytokine secretion in human bladder, laryngeal and intestinal epithelial cell lines, depending on the anatomical derivation of the cells or the inflammatory status [35]. Other studies reported that Nef induces proliferation and de-differentiation of podocytes, suggesting that it may have a critical role in the pathogenesis of HIV-associated nephropathy [36]–[39]. The present data indicate that Nef, targeting EC, may also play a crucial role in AIDS-related gastrointestinal dysfunction.

So far, most of the activities of Nef have been attributed to the intracellular expression of the protein or its association with virions. However, Nef is known to be secreted from infected cells [16] in association with small membrane-bound vescicles [17], [18].

We found that purified exogenous Nef is efficiently taken up by unstimulated and IFN-γ stimulated Caco-2 cells and, independently from IFN-γ treatment, Nef appeared as a diffuse intracytoplasmatic positivity. Interestingly, in IFN-γ-treated Caco-2 cells, internalized Nef was still retained after 24 h of treatment suggesting that IFN-γ may prolong Nef up-take or its intracellular survival.

Fuji et al reported that a high percentage of sera from HIV-1-infected individuals contained soluble Nef at a concentration of 1–10 ng/ml, while patient's sera in which Nef was not detectable contained high titers of anti-Nef antibodies [16]. This concentration may be higher in the gut-associated lymphoid tissue (GALT) where virion-trapping dendritic cells, as well as virion-infected CD4^+^ T cells and macrophages, are densely packed [40]. Thus, the effective Nef concentration for epithelial cell impairment could be reached *in vivo*. Notably, our results were confirmed with a lower dose of native Nef protein secreted in the supernatants of Nef transfected 293T cells.

Ndolo et al [41] reported that GALT is an early site for strong positive selection for functional full-length Nef. GALT is an early site for active viral replication and may be the major viral reservoir, even in patients receiving HAART. The selective loss of intestinal CD4^+^ T cells is likely to explain, together with mucosal barrier breakdown, the preponderance of opportunistic infections at mucosal sites [42]. Thus, the study of the events occurring at mucosal sites are relevant to a better understanding of AIDS pathogenesis and to help the development of preventive or therapeutic vaccines against HIV that may be administered by the mucosal route. The goal would be to prevent or reduce the propagation of HIV at mucosal surfaces to restore the immunological and epithelial integrity of the mucosal barrier and to block pathways through which microbial products cause systemic immune activation [43], [44]. In this context, our data may provide new directions for therapeutic interventions. Notably, the Nef-induced increase in intestinal permeability might favour its uptake during immunization procedure, while its ability to partially restore epithelial barrier integrity in the presence of IFN-γ and to prevent the IFN-γ-induced apoptosis might counteract gastrointestinal dysfunction in AIDS patients.

Besides their barrier function, intestinal EC participate in mucosal immune and inflammatory reactions secreting cytokines and chemokines. In health, the GI tract maintains a balance between immune tolerance and rapid responsiveness [45], [46], which is perturbed in HIV infection [4].

It is well known that Nef induces cytokine and chemokine secretion by monocytes/macrophages [47]–[49] and DC [19] interfering with cellular signal transduction pathways [12]. This study provides evidence that intestinal EC respond to Nef, producing increased level of TNF-α, IL-6 and MIP-3α and decreased level of IL-10. Cytokine release, as well as the down-regulation of IL-10 production, might favour activation of T cells, rendering them permissive for the replication of the virus [50] and activating HIV-1 replication in latently infected cells [51]. In addition, we found that Nef inhibits the IFN-γ induced AA cascade. Caco-2 cell differentiation is characterized by an increase in polyunsatured fatty acids, such as AA, critical in immunity and inflammation [52], [53]. We demonstrated that Nef abrogated the IFN-γ-induced up-regulation of PGE_2_ release, by a reduction of COX-2 expression level. The IFN-γ-induced up-regulation of PGE_2_ release was not associated with an increased COX-2 expression, suggesting that IFN-γ might increase COX-2 activity instead of its expression levels or might act further downstream, exerting a positive regulatory role at the level of prostaglandin E synthase expression.

These results may be relevant, considering that local inflammatory response may be both harmful and helpful for viral dissemination. While recruiting HIV-susceptible CD4^+^ T cells to the site of infection, cytokines/chemokines also lead to increased recruitment of HIV-specific CD8^+^ T cells. Thus, in the early step after viral transmission, HIV-1 might benefit from the balance between inflammation and protective immunity induced by Nef, creating a microenvironment that favours viral replication.

Taken together, these findings suggest that Nef upsets the IFN-γ-induced impairment of intestinal epithelial cells, possibly prolonging cell survival and allowing the accumulation of viral particles before cell destruction and virus release. This is relevant considering that most viruses have evolved strategies to defend themselves against host IFN responses [54].

A better understanding of the role of Nef in HIV-1-related intestinal dysfunction will help in the development of novel strategies to provide protection against the first exposure to the virus and against the subsequent long-lasting mucosal damage.
